# Initiation and elongation factor co-expression correlates with recurrence and survival in epithelial ovarian cancer

**DOI:** 10.1186/s13048-022-00998-y

**Published:** 2022-06-19

**Authors:** Monika Sobočan, Daniela Brunialti, Sussanne Sprung, Christoph Schatz, Jure Knez, Rajko Kavalar, Iztok Takač, Johannes Haybaeck

**Affiliations:** 1 Department of Pharmacology, Faculty of Medicine, University of Maribor, Maribor, Slovenia; 2 Department of Obstetrics and Gynecology, Faculty of Medicine, University of Maribor, Maribor, Slovenia; 3 Division of Gynecology and Perinatology, University Medical Centre Maribor, Maribor, Slovenia; 4 Institute of Pathology, Neuropathology and Molecular Pathology, Medical University of Innsbruck, Innsbruck, Austria; 5 Department of Pathology, University Medical Centre Maribor, Maribor, Slovenia; 6 Diagnostic & Research Center for Molecular Biomedicine, Institute of Pathology, Medical University of Graz, Graz, Austria

## Abstract

High grade epithelial ovarian cancer (EOC) represents a diagnostic and therapeutic challenge due to its aggressive features and short recurrence free survival (RFS) after primary treatment. Novel targets to inform our understanding of the EOC carcinogenesis in the translational machinery can provide us with independent prognostic markers and provide drugable targets. We have identified candidate eukaryotic initiation factors (eIF) and eukaryotic elongation factors (eEF) in the translational machinery for differential expression in EOC through *in-silico* analysis. We present the analysis of 150 ovarian tissue microarray (TMA) samples on the expression of the translational markers eIF2α, eIF2G, eIF5 (eIF5A and eIF5B), eIF6 and eEF1A1. All translational markers were differentially expressed among non-neoplastic ovarian samples and tumour samples (borderline tumours and EOC). In EOC, expression of eIF5A was found to be significantly correlated with recurrence free survival (RFS) and expression of eIF2G and eEF1A1 with overall survival (OS). Expression correlation among factor subunits showed that the correlation of eEF1A1, eIF2G, EIF2α and eIF5A were significantly interconnected. eIF5A was also correlated with eIF5B and eIF6. Our study demonstrates that EOCs have different translational profile compared to benign ovarian tissue and that eIF5A is a central dysregulated factor of the translation machinery.

**Supplementary Information:**

The online version contains supplementary material available at 10.1186/s13048-022-00998-y.

## Introduction

Ovarian cancer (OC) represents the most lethal gynaecological cancer with an annual incidence of 230,000 women diagnosed with epithelial OC (EOC) [1]. Ninety-five percent (%) of OCs are EOC [2]. Recent data emphasizes the importance of understanding molecular mechanisms and histological subtypes of OC as different subtypes and clinicopathological features form distinct disease features [2–4]. There have been several proposed signalling pathways in OC. According to the Cancer Genome Atlas (TCGA) one of the most frequently activated signalling pathways (in approximately 60% of all OCs) is the hyperactivation of phosphoinositol 3 kinase (PI3K)/protein kinase B (AKT)/mammalian target of rapamycin (mTOR) (PI3K/AKT/mTOR) pathway. This pathway is involved in cancer cell growth, survival, metabolic programming, autophagy, transcription regulation, and angiogenesis [5]. However, molecular studies found no specific mTOR target that pointed to statistically significant clinical outcomes for patients treated with common mTOR inhibitors [6]. The anwser might be that in order to appropriately target this pathway, more downstream markers should be investigated. Therefore an important target could be the process of protein synthesis. Protein synthesis depends largely on the ability and efficacy of the process of translating mRNAs into protein. The translation process is divided into initiation, elongation and termination. Eukaryotic initiation factors (eIFs) facilitate the translational process through the mRNA binding to the 40S ribosomal subunit [7]. The dysregulation of protein synthesis has been associated with carcinogenesis and several reports show, that the alteration of initiation and elongation pathways was implicated in worse outcomes in different cancer subtypes. The mechanisms of action through which protein synthesis develops are many and not fully understood [8].

Downstream the cascade of PI3K/Akt/mTOR signalling, mTOR expression in tissue was significantly related to eIF-4E tissue expression and the serous histological subtype in one of the first studies addressing OC eukaryotic initiation factors (eIF) expression. Overexpression of the eIF-4E was shown in that study to have better overall survival (OS) [9]. Previously single eIFs have been investigated in OC. The factor eIF-5A2 was not detectable in normal ovarian tissue, in 7% of cystadenomas, 30% of borderline tumours (BLTs) and 53% of OCs [10]. Growth was restricted in cell lines in which antisense DNA inhibited eIF-5A2. The presence of eIF-5A2 overexpression in tissue microarrays (TMAs) also showed an association with advanced OC stage [10, 11], ascending tumour grade and increased rates of Ki-67 [10]. Expression of eIF5A2 in cell lines was also significantly higher in tumors that spread peritoneally and eIF5A2 knockdown reduced stem-cell related markers, indicating that eIF5A2 inhibition might be important in the OC cell self-renewal ability. This data translated also to eIF5A2 overexpression in tissue to shortened patient survival [10]. Also when those lines were treated by chemotheraputic agents, the inhibition of eIF5A2 lead to improved chemosensitivity [12]. Previous studies in eIF6 and OC showed attenuation of the expression can contribute to slowing down the cell cycle, but not reduce migration and invasion by using Notch-1 signalling inhibition [13]. Additional in vitro evaluation showed, that selective Cdc42 inhibition could stop cell migration and invasion in a cell line with overexpressed eIF6 [14]. Additionally the elongation factors (eEF) 1A2 showed oncogenic properties such as focus formation, increase in growth rates of tumours and fibroblast cancer trnasformation [15]. This was later supported by the understanding that eEF1A2 interacts with the tumour suppressor protein p16 (INK4a) and through this dysregulated mechanism supports cancer proliferation [16]. Currently there are more than 12 known eIFs (eIF1, eIF1a, eIF2, eIF2b, eIF3, eIF4a, eIF4e, eIF4g, eIF4b, eIF4h, eIF5 and eIF5b and eIF6) [17]. In elongation, the key components are two main types of eukaryotic elongation factors (eEF). These types are eEF1 an eEF2 [18]. Individual promising reports show that eIFs and eEF may have a role in OC, but there is still no clear understanding of the role different markers together in the translational framework of OC. The aim of this research was to elucidate the role of eIFs and eEFs in high grade EOC and their potential interplay in carcinogenesis.

## Methods

### Sample selection

We have identified patients with EOC, BLTs and women which have undergone risk reducing salpingo-oophorectomies with a benign pathological outcome.

Tumour tissue samples were identified in the University Medical Centre Maribor, Division of Gynaecology and Perinatology database for OC treated between the January 2009 to December 2014. The inclusion criteria for this study were patients from age 18 years and above with a diagnosis of OC. To be included into the analysis, formalin-fixed paraffin embedded (FFPE) tumour tissue samples had to be available. Patients samples were excluded from the study if there was chemotherapy administrated prior to surgery or diagnostic ovarian tumour biopsy. Clinical data was retrieved from the electronic medical patient records. The available data included age, FIGO tumour stage 1988 [19], date of disease diagnosis, modality of primary treatment, adjuvant systemic therapy, date of first disease recurrence as well as date of death.

All tissue samples were reviewed for inclusion by two board certified expert gynaecologic pathologist (RK, SS). Only tumours with sufficient adnexal tissue availability for staining as determined by the pathologist were included in this study. This study was performed in accordance to the National Medical Ethics Committee of Slovenia Review Board approval (registration number 0120–565/2019/4).

### Bioinformatic eIF subunit selection 

The mRNA TCGA OC (OV) dataset of 602 samples was analyzed *in-silico* (data source: http://gdac.broadinstitute.org/). Groups were built based on Grades as stated in the clinical information. G2 versus G3 revealed a significantly differently (*p* < 0.05) expression of *EEF1A1* using the R function wilcox.test. G3 compared to GB (BLT) showed a significantly differently expression of *EIF2S1* (*EIF2α* synonym), *EIF2S3* (*EIF2G* synonym) and *EIF5* (*EIF5A* synonym). *EEF1A1* additionally reached a high AUC. *EEF1A1*, *EIF2A*, *EIF2G* and *EIF5A* combined with *EIF6* from literature and *EIF5B* in relation to *EIF5* were used for further analyses on protein level in-vitro. Antibodies were established for the translation factors EIF1A1, EIF2, EIF2G, EIF5A, EIF5B and EIF6.

### Sample preparation and evaluation

Tumour samples were evaluated by two expert pathologists (SS, JH) and relevant tumour areas were identified. After identification tissue arrays (1.5 mm in diameter) were punched out. From the retrieved tissue, tissue sections were cut (4 μm) and fixated for immunohistochemical staining.

Immunohistochemical staining was performed using a Ventana Immunostainer XT (Ventana Medical Systems, Tucson, AZ, USA), using an ultra-VIEW Universal DAB Detection Kit (Ventana Medical Systems, Tucson, AZ, USA) and cell conditioning solution for 30 min using heat-induced epitope retrieval (HEIR). Staining was performed for the subunits eEF1A1 (rabbit, monoclonal, AB157455, Abcam, Cambridge, UK), eIF2α/2S1 (rabbit D7D3 5324, monoclonal, Cell Signaling, Danvers, USA), eIF2G (rabbit, polyclonal, AB225953, Abcam, Cambridge, UK), eIF5A (rabbit, polyclonal PA5–29204, Invitrogen, Carlsbad, Germany), eIF5B (rabbit, polyclonal, AB251824, Abcam, Cambridge, UK) and eIF6 (rabbit polyclonal A303-030A-M, Bethyl/Biomol, Montgomery, USA).

**Fig. 1:**
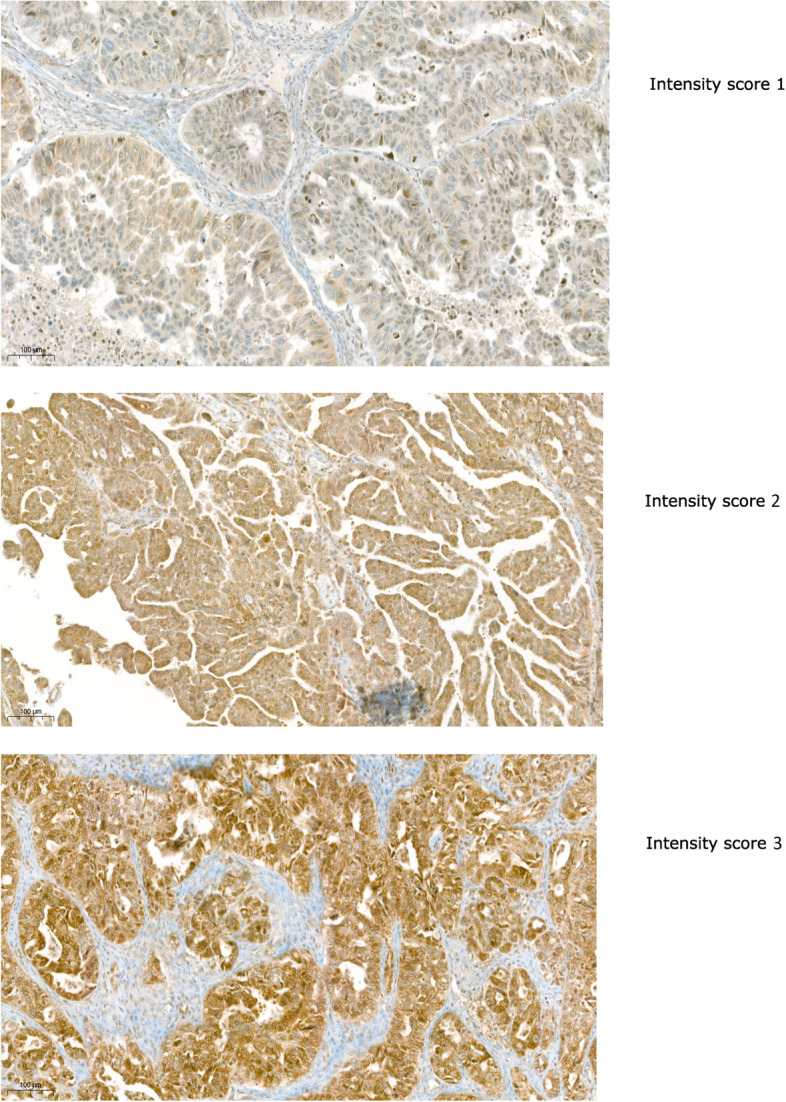
Intensity scores for eIF5A

The tissue sections were evaluated based on visual estimation on staining density and staining intensity in the following components: nucleus, cancer stroma, normal stroma and cytoplasm. Density was scored from one to four, according to the estimated percentage of stained cells (0–25% =1, 25–50% =2, 50–75% =3, 75–100% =4) and staining intensity was scored from zero to three (0 = negative, 1 = weak, 2 = moderate, 3 = strong). A sample of intesity staining is represented in Fig. 1. A combined score of intensity and density was calculated by multiplication of the individual scores. Further evaluation was performed using the combined score (CS). The maximum value of CS was 12. If the staining quality was poor and the sample could not be successfully evaluated, the sample was excluded from further statistical analysis.

### Statistical analysis

Continous variables were expressed as median variables (standard deviation) and proportions were reported as percentages. Combined staining scores were anayzed using a non-parametric test (Wilcoxon Rank Sum Test) to compare groups. The correlation analysis was done using Spearman’s rank correlation. Survival analysis was performed using Kaplan-Meier analyses, through the univariate survival analysis (log-rank test). Statistical significance was set at *p* < .05. All analyses were performed using SPSS for Mac Version 23.0 (IBM Corp., Armonk, NY, USA).

## Results

### Cohort characteristics

We have analysed 75 high grade EOC samples, 43 BLTs (22 mucinous tumours, 19 serous tumours and 2 of mixed histology) and 32 samples of healthy ovarian tissue procured through risk-reducing surgery.

### Expression profiles of translational factor subunits

Healthy ovarian tissue was stained for the translation factor subunits eIF2α, eIF2G, eIF5A, eIF5B, eIF6 and eEF1A1. The subunit expression in healthy ovarian tissue was compared against expression profiles of BLT tissue and EOC tissue. There were statistically significant differences between healthy ovarian tissue, BLT tissue and EOC (Supplemental data Table 1).

Further exploration of differences in subunit expression between EOC and BL tumours (Table 1) revealed statistically significant changes in individual subunit expression. BL tumours showed a significant overexpression of eIFs in comparison to EOC. After adjusting for within group differences between serous and mucinous BLTs, eIF5A was significantly overexpressed in the cancer stroma and cytoplasm of BL. Cancer stroma exhibited significant differences in overexpression also for the subunits eIF6. No comparison of expression was possible for eEF1A1, eIF2α, eIF2G, eIF5A, eIF5B and eIF6 normal stroma expression due to the BLT staining failure. The combined expression score for all tumours (EOC and BL) in eEF1A1, eIF2α, eIF5B and eIF6 nuclear expression was equal (CS = 0).

**Table 1:** Subunit combined score comparison among borderline tumors (BLTs) and epithelial ovarian cancer (EOC) samples

		BLT subtype	BLT subtype (serous vs. mucinous BLT) mean CS (SD)	BLT subtype (serous vs. mucinous BLT) difference (*p*-value)	Mean CS (SD)	BLT vs. EOC expression significance
eIF5A cancer stroma	EOC (*n* = 74)	/	3.9 (1.9)	/	3.9 (1.9)	U = 1077; *p* < .003
	BLT (*n* = 41)	mucinous	5.6 (2.1)	.402	5.4 (2.5)	
		serous	5.2 (2.0)			
eIF5A nuclear	EOC (*n* = 75)	/	10.7 (3.4)	/	10.7 (3.4)	U = 1506; *p* < .273
	BLT (*n* = 41)	mucinous	11.4 (1.8)	.685	11.4 (2.2)	
		serous	11.4 (2.7)			
eIF5A cytoplasm	EOC (*n* = 75)	/	4.1 (2.6)	/	4.1 (2.6)	U = 974; *p* < .000
	BLT (*n* = 41)	mucinous	5.8 (2.5)	.360	6.2 (2.7)	
		serous	6.6 (3.0)			
eIF5B cancer stroma	EOC (*n* = 73)	/	2.1 (1.5)	/	2.1 (1.5)	U = 1023; *p* < .001
	BLT (*n* = 41)	mucinous	3.7 (1.7)	.004	3.0 (1.6)	
		serous	2.3 (1.2)			
eIF5B cytoplasm	EOC (*n* = 74)	/	1.8 (1.9)	/	1.8 (1.9)	U = 919; *p* < .000
	BLT (*n* = 41)	mucinous	4.7 (3.3)	.036	3.7 (2.7)	
		serous	2.6 (1.3)			
eIF6 cancer stroma	EOC (*n* = 75)	/	2.3 (1.0)	/	2.3 (1.0)	U = 1142; *p* < .005
	BLT (*n* = 41)	mucinous	3.0 (1.1)	.643	2.9 (1.1)	
		serous	2.8 (1.1)			
eIF6 cytoplasm	EOC (*n* = 75)	/	6.24 (2.7)	/	6.24 (2.7)	U = 1311; *p* < .066
	BLT (n = 41)	mucinous	6.6 (2.6)	.196	7.1 (2.7)	
		serous	7.9 (2.7)			
eIF2G cancer stroma	EOC (*n* = 75)		3.2 (1.8)	/	3.2 (1.8)	U = 842; *p* < .000
	BLT (*n* = 41)	mucinous	5.5 (2.1)	.044	5.0 (2.2)	
		serous	4.1 (2.0)			
eIF2G nuclear	EOC (*n* = 75)		5.9 (2.5)	/	5.9 (2.5)	U = 1183; *p* < .009
	BLT (*n* = 41)	mucinous	6.1 (2.4)	.013	7.3 (3.0)	
		serous	8.5 (3.2)			
eIF2G cytoplasm	EOC (*n* = 75)	/	5.7 (2.3)	/	5.7 (2.3)	U = 1192; *p* < .010
	BLT (*n* = 41)	mucinous	6.1 (2.4)	.041	7.0 (2.7)	
		serous	7.9 (2.9)			
eIF2α cytoplasm	EOC (*n* = 75)	/	9.5 (2.4)	/	9.5 (2.4)	U = 1608; *p* < .978
	BLT (*n* = 41)	mucinous	8.6 (2.4)	.034	9.5 (2.3)	
		serous	10.2 (2.0)			
eIF2α cancer stroma	EOC (*n* = 75)	/	3.1 (1.1)	/	3.1 (1.1)	U = 1573; *p* < .815
	BLT (*n* = 41)	mucinous	3.3 (0.9)	.102	3.1 (3.6)	
		serous	2.7 (1.1)			
eEF1A1 cytoplasm	EOC (*n* = 75)	/	9.0 (2.7)	/	9.0 (2.7)	U = 1546; *p* < .675
	BLT (*n* = 41)	mucinous	8.0 (1.8)	.002	8.9 (2.1)	
		serous	10.0 (2.0)			
eEF1A1 cancer stroma	EOC (*n* = 72)	/	4.3 (3.4)	/	4.3 (3.4)	U = 1059; *p* < .004
	BLT (*n* = 41)	mucinous	7.2 (2.6)	0.001	6.0 (3.0)	
		serous	5.0 (3.6)			

### Clinical outcomes of patients with EOC

The mean age of the cohort was 61.0 (SD 11.2). The follow up period was 72 months. Sixty-one percent of women (*n* = 46) suffered disease recurrence during the follow up period. Mean recurrence free survival (RFS) was 33.9 months (CI 95 28.3 months – 39.6 months). Disease specific death occurred in 68% (*n* = 51) women. Mean OS was 43.5 months (CI 95 37.5 months – 49.0 months). Statistically significant subunits (Table 2) in the primary analysis were further evaluated in regard to the impact factors had on RFS and OS. Based on median scoring values to categorize subunits as high or low expressed were designated (Supplemental data Table 2).

**Table 2:** Significance of subunit expression on recurrence free survival (RFS) and overall survival (OS)

		Number of evaluated *samples*	CS score (SD)	RFS	OS
eIF6	eIF6 cytoplasm	75	6.24 (2.7)	U = 640; *p* < .742	U = 600; *p* < .876
	eIF6 nuclear	75	N/A	N/A	N/A
	eIF6 normal stroma	60	2.2 (1.0)	U = 372; *p* < .638	U = 278; *p* < .410
	eIF6 cancer stroma	75	2.3 (1.0)	U = 648; *p* < .821	U = 599; *p* < .872
eIF5B	eIF5B cytoplasm	74	1.8 (1.9)	U = 597; *p* < .525	U = 566; *p* < .685
	eIF5B nuclear	74	N/A	N/A	N/A
	eIF5B normal stroma	50	1.96 (1.3)	U = 236; *p* < .305	U = 228; *p* < .749
	eIF5B cancer stroma	73	2.1 (1.5)	U = 483; *p* < .059	U = 536; *p* < .507
eIF5A	eIF5A cytoplasm	75	4.1 (2.6)	U = 531; *p* < .102	U = 469; *p* < .073
	eIF5A nuclear	75	10.7 (3.4)	U = 546; *p* < .025	U = 503; *p* < .036
	eIF5A normal stroma	57	4.3 (1.9)	U = 332; *p* < .341	U = 310; *p* < .510
	eIF5A cancer stroma	74	3.9 (1.9)	U = 481; *p* < .043	U = 446; *p* < .048
eIF2G	eIF2G cytoplasm	75	5.7 (2.3)	U = 562; *p* < .209	U = 463; *p* < .063
	eIF2G nuclear	75	5.9 (2.5)	U = 608; *p* < .481	U = 439; *p* < .031
	eIF2G normal stroma	52	4.2 (1.9)	U = 261; *p* < .314	U = 247; *p* < .466
	eIF2G cancer stroma	75	3.2 (1.8)	U = 647; *p* < .820	U = 482; *p* < .122
eEF1A1	eEF1A1 cytoplasm	75	9.0 (2.7)	U = 553; *p* < .177	U = 516; *p* < .235
	eEF1A1 nuclear	75	N/A	N/A	N/A
	eEF1A1 normal stroma	54	3.3 (2.7)	U = 308; *p* < .424	U = 278; *p* < .379
	eEF1A1 cancer stroma	72	4.3 (3.4)	U = 573; *p* < .611	U = 387; *p* < .021
eIF2α	eIF2α cytoplasm	75	9.5 (2.4)	U = 629; *p* < .640	U = 581; *p* < .771
	eIF2α nuclear	75	N/A	N/A	N/A
	eIF2α normal stroma	66	2.8 (1.6)	U = 443; *p* < .260	U = 612; *p* < .086
	eIF2α cancer stroma	75	3.1 (1.1)	U = 598; *p* < .425	U = 589; *p* < .710

**Fig. 2:**
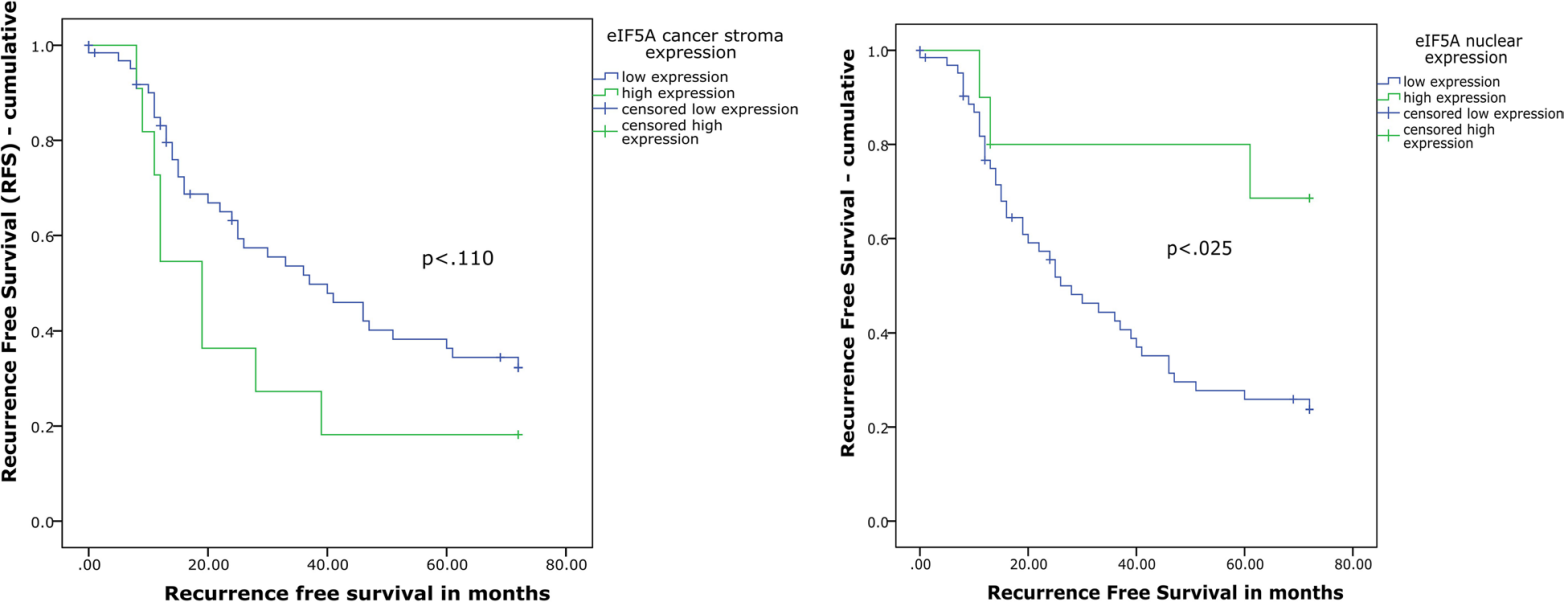
Survival plot of recurrence free survival (RFS) for significant translational subunits

Primary factor analysis showed a statistically significant difference in RFS for the subunit eIF5A in nuclear factor expression and in cancer stroma expression. The nuclear expression remained statistically significant (*p* < .025) for eIF5A. Mean survival in high eIF5A nuclear expression was 58.7 months (CI95 44.1–73.4 months) and 35.6 months (CI95 29.1–42.2 months) in low eIF5A nuclear expression (Fig. 2). The eIF5A cancer stroma expression was not statistically significant in RFS. Mean survival in low eIF5A cancer stroma expression was 41.1 months (CI95 34.2–48.0 months) and 27.4 months (CI95 13.9–41.0 months) in eIF5A cancer stroma high expression.

**Fig. 3:**
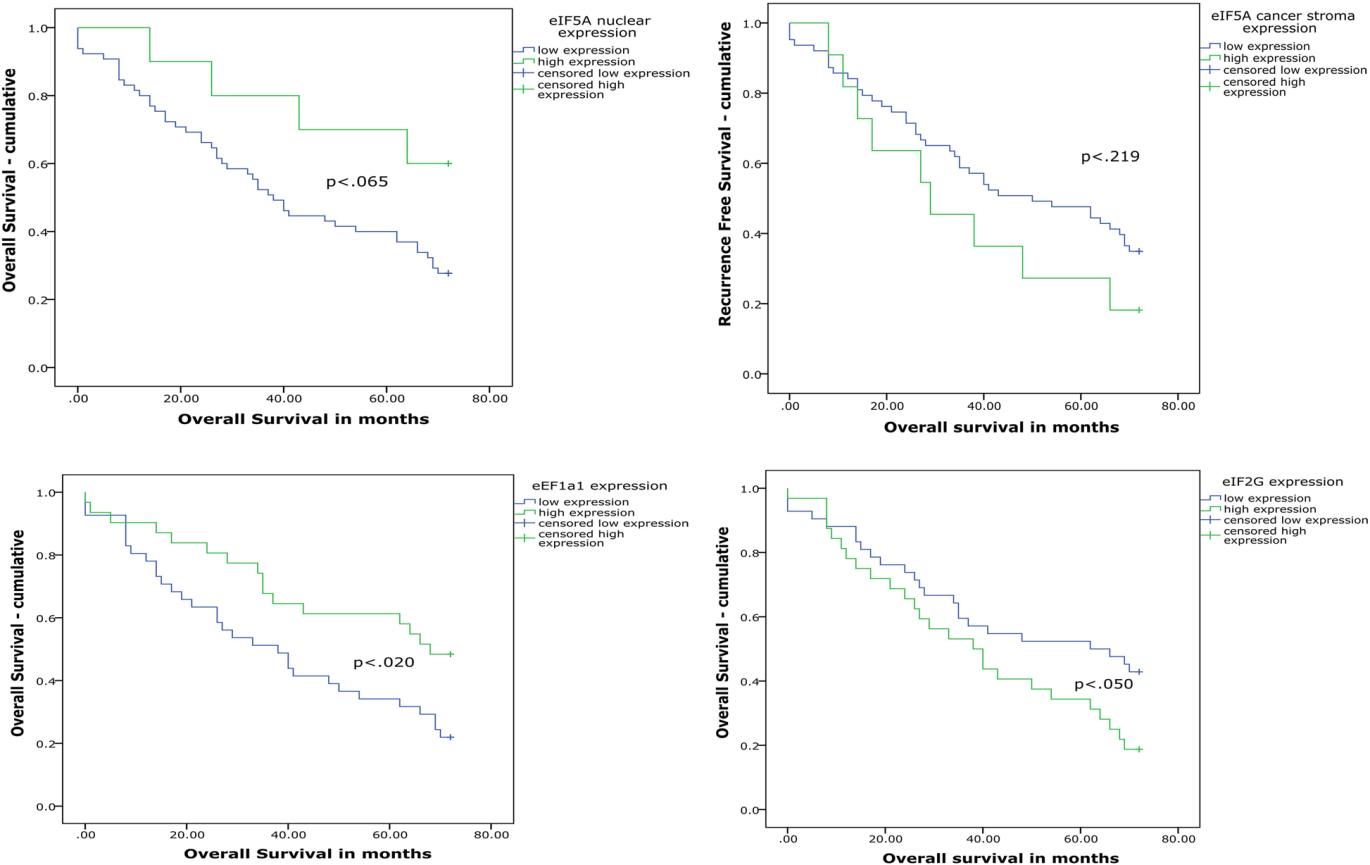
Survival plot of OS in significant translation subunits

OS remained significantly altered with differences in expression of eIF2G (*p* < .050) and eEF1A1 (*p* < .020), but not for eIF5A cancer stroma expression (*p* < .219) or eIF5A nuclear expression (*p* < .065) (Fig. 3). eIF5A nuclear expression was 41.3 months (CI95 34.8–47.7 months) in low expression and 57.9 months (CI95 44.9–70.9 months) in high expression levels. The eIF5A cancer stroma expression levels showed that OC with low levels of eIF5A cancer stroma expression had an OS of 45.4 months (CI95 38.9–52.0 months) and 36.5 months (CI95 22.7–50.4 months) in high levels of expression in eIF5A cancer stroma expression. Survival analysis showed that low expression levels of eIF2G were significantly connected with OS. OS in low expression of eIF2G was 47.4 months (CI95 39.3–55.5 months) and 39.8 months (CI95 31.2–48.3 months) in the high expression group. High levels of elongation factor eEF1A1 expression were shown to be significantly connected to increased OS 52.0 months (43.2–60.9 months). Low expression levels of eEF1A1 were correlated with worse survival of a mean value of 38.8 months (30.8–46.7 months).

Final analysis showed, that the markers eIF5A nuclear expression should be further assessed in evaluating RFS and eIF2G and eEF1A1 for OS in OC.

### EOC subunit co-expression analysis

**Fig. 4:**
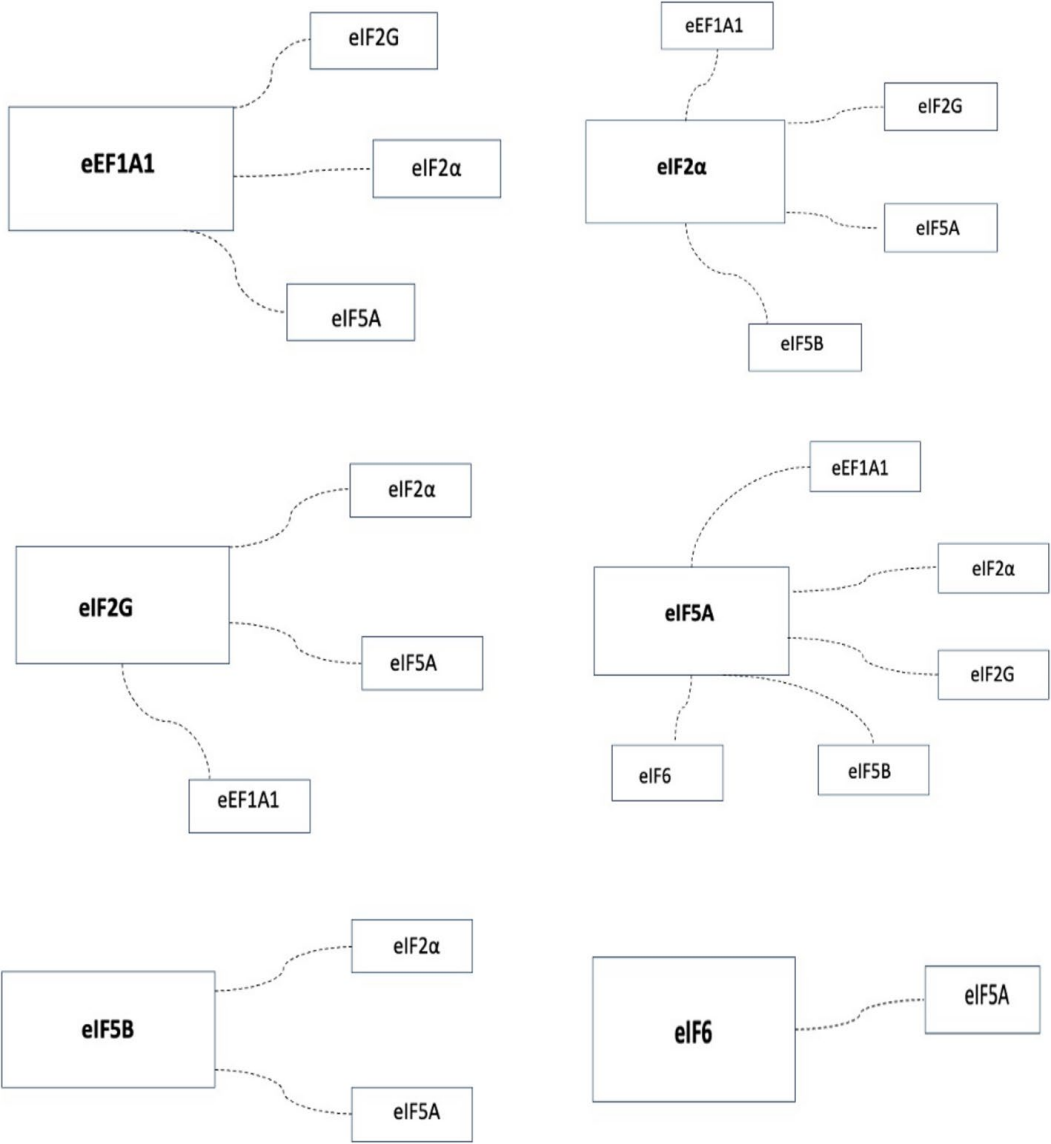
Translational subunit associations based on the Spearman rank correlation analysis

There were significantly correlated expression patterns among subunits (Supplemental data – Table 3). Figure 4 represents the correlations among different subunits.

## Discussion

Our assessment of translational markers showed, that across all tested subunits expression profiles were significantly different in normal ovarian tissue, compared to BLTs and high grade EOC. Clinical outcomes were significantly worse for women with low levels of eIF5A nuclear expression, but not if women had high levels of eIF5A cancer stroma overexpression. The differences in eIF5A expression did not translate in impact on OS in women with EOC. OS was significantly impacted if overexpression of eIF2G was present and if low levels of eEF1A1 occurred.

Translational regulation has been proposed to be a key element of adaptation to stress conditions in carcinogenesis. These events occur as a response to the tumour microenvironment, immunological changes and their proliferation [20]. The subunits involved in the translational machinery of OC in tumour tissue or cell lines has been reported for eIF6 [13, 14, 21, 22], eIF5A [23–26], eIF5B [27], eEF1 [28–30] and eIF2α [31–36]. No reports were found to have directly assessed eIF2G (or synonym eIF2S3) for OC. In our study the co-expression analysis showed, that the eIF2G and eEF1A1, eIF5A and eIF2α were significantly interconnected. Therefore further work understanding the translational machinery should involve multimodal approaches to the assessment of several eIF and eEF units.

OS was significantly impacted by overexpression of eIF2G in our study. Interestingly, while eIF2G was found to be present in different tissues, little is known on how it affects carcinogenesis. Recent reviews interconnect eIF2G to cancer/testis antigens (C/T antigens) which are able to bypass immune response in patients in whose cancers express these antigens. It was hypothesized that carcinogenesis might be part of the ability to evade immune response if overexpression of eIF2G is present in ovarian tumours [37]. However, more mechanistic studies in OC need to be performed to elucidate its role in the process of carcinogenesis in high-grade EOC.

Previous studies have established that an overexpression of eIF5A was connected to worse overall outcomes in different cancer subtypes [38]. eIF5A has two isoforms, eIF5A-1 and eIF5A-2 which are expressed also during carcinogenesis and are in human up to 84% identical [39]. The biological function of eIF5A-1, which was stained in our study, is connected to histogenesis in most cells. eIF5A-2 however was found only in cancer cells [39]. Although eIF5A-1 has been deemed as a eukaryotic initiation factor, it has an important role in elongation as studies in the depletion of eIF5A-1 show the cessation of ribosome activity at many sequences. Evidence also shows, that by silencing the expression of eIF5A-1, ribosomes accumulate at stop codons and 3’UTR, suggesting a defect in translation termination [40]. Evaluating eIF5A-2 was shown to be prognostic for OC in previous research [10, 26] and has been connected with RFS and OS. Early cell line analysis showed, that eIF5A is connected with cell survival [25]. Our study showed that in EOC the eIF5A-1 expression in the cytoplasm and stroma was higher than in BLTs and it was significantly different from the expression in healthy ovarian tissue, but we did not show a significant correlation with RFS or OS through our survival analyses. The two isoforms of eIF5A are on different chromosome locations and while eIF5A-1 is crucial in elongation and RNA metabolism [41, 42], eIF5A-2 is tissue and even cell-type specific and was shown to be involved in carcinogenisis previously. Our analysis adds that when evaluating eIF5A expression in EOC should involve analysis of eIF5A-1 and also eIF5A-2.

Moving downstream in the evaluation of the expression landscape, the elongation factor eEF1A1 has been shown to be significantly correlated with OS. This elongation factor has two subunits, eEF1A1 and eEF1A2. eEF1A2 has been extensively studied in OC and was shown to contribute to cell proliferation and worse outcomes if overexpressed [43]. eEF1A1 is involved in the regulation of the cytoskeleton and also in the control of cell proliferation and death [43]. Interestingly, it has been reported, that the presence of eEF1A1 and eEF1A2 in most normal cells is mutually exclusive [44]. Improved understanding on how the relation of eEF1A1 towards eEF1A2 in OC is will offer us also better abilities to use this marker, which showed significant correlation with OS, for prognostic purposes.

Our data analysis did not show a significant correlation of clinical outcomes with eIF2α and eIF6. We did not confirm data from previous groups showing that eIF6 underexpression in ovarian tumour tissue to worse RFS and OS [21]. Investigation in OC cell lines [13, 14] showed, that eIF6 expression was connected to motility and tumour metastasis. Interestingly miRNA analysis further did not show the connection between eIF6 the component Dicer and diregulation in recurrent OC [22]. Therefore the data on this marker is currently still conflicting and will need further evaluation also in accordance with the correlation profiles of eIF5B and eIF5A.

We evaluated cytoplasmic and nuclear expression of different eIFs and eEFs. It has been shown previously that the phosphorylation of eIF4E in its nuclear component represents a major impact on mRNA transport [45]. Furthermore, eIF2α phosphorylation has also been established to influence mRNA containing open reading frames (ORFs) in 5′ untranslated regions (5’UTR) [46]. Tejada et al. [47] evaluated the location of subunits in brain tumours where it was shown that localization of subunit expression was connected to specific cell subtypes. eIF5A nuclear expression was significantly correlated with RFS. Significant correlation was not present in cytoplasmic expression of eIF5A. Substantal work has been done on understanding impact of eIF5A localization on cellular pathophysiology. eIF5A has been reported to be the only protein containing hypusine and the subunit is activated by post-translational synthesis of hypusine [48]. It has been proposed that hypusinated eIF5A is a RNA binding protein associated with exportins [49]. Interestingly hypusinated eIF5A, which impacts protein synthesis was shown to be mainly localized in the cytoplasm [48]. It is still unclear what the role of eIF5A in the nucleus is, potentially warranting the hypothesis that nuclear localization is a prerequisite for abnormal cytoplasmic protein activation.

Significant expression of eEF1A was localized in the cancer stroma and interconnected with OS. Subunits of eEF1A have been demonstrated in human lung cancer in the nucleus as well as in the cytoplasms [50]. In order to further determine the functional impact localization has on the outcome of ovarian cancer further eEF1 subunit co-expression would need to be analzyed. Up to now there are no additional reports on the impact eIF localization has on eF2G.

Studies on eIF and eEF subunit expression have been mostly performed on samples of women with epithelial serous ovarian cancer. Only a small proportion of women in Ali-Fehmi et al. were of endometrioid, clear cell or mucinous histology [23]. No subset analysis was available in this study to determine the impact of different histologies on eIF5A expression. Other studies for eIF5A were performed on cell lines, not enabling clear evaluation of histotype impact on eIF5A expression. There are currently no reports on eIF2G in ovarian cancer available. For eEF1A, interestingly, a gene expression study on non-epithelial ovarian cancer (granulosa cell tumor, mixed germ cell tumor, yolk sac tumour, immature teratoma, malignant mature teratoma, dysgerminoma, thecoma and juvenile granulosa cell tumor) was performed to compare expression with normal ovarian tissue. It was shown that cytoplasmic expression of eEF1A was increased in non-epithelial ovarian cancer [28]. Furthermore, it was shown that eEF1A2 overexpression was present in 75% of clear cell carcinomas, which is higher than in other histological subtypes [29]. This calls for further exploraton of the role eEF1A has in non-epithelial cancers and its correlation to epithelial cancer.

The significant markers eIF5A, eIF2G and eEF1A have not been clearly correlated to clinical parameters of prognostic value in ovarian cancer. A gap in understanding the role of prognostic markers and the significant eIF subunits needs to be explored further in the future.

This study has some limitations which need to be taken into consideration. The translational machinery is a complex process, and while the available factors address important aspects of it, not all subunits currently known were analysed. Our study however offers with the involvement of six subunits a unique evaluation of their interconnected relations and impact on outcomes. The outcomes of this research are based on IHC data and should be studied further in order to correclty elucidate the mechanisms of action in OC.

## Conclusion

Translational subunits in OC and BLTs are differentially expressed in comparison to normal ovarian tissue. The evaluation of specific subunits in OC, such as eIF5A, eIF2G and eEF1A can serve as a tool to evaluate tumour agressiveness and enable the use of this markers to further investigate and determine their potential of druggable targets. The understanding of translational biology in OC needs to move towards a more holistic aproach of integrating different eIF and eEF subunits into analysis as they have been shown to be interconnected in our study. Thus, they need to be understood as individual biomarkers as well as in their interconnectedness in order to achieve knowledge on significant therapeutic targets in OC.

## Supplementary Information

**Additional file 1.**
