# Supplementary material for: Initiation and elongation factor co-expression correlates with recurrence and survival in epithelial ovarian cancer
Source: J Ovarian Res. 2022 Jun 19;15:73. doi: 10.1186/s13048-022-00998-y (PMC9208098; doi:10.1186/s13048-022-00998-y)
Supplement: Supplementary file 1 — Additional file 1. [file 13048_2022_998_MOESM1_ESM.docx]

Supplemental data Table 1

| **Table 1: Subunit combined score comparison between normal ovarian tissue and borderline tumors (BLTs) or epithelial ovarian cancer (EOC) samples** | | | | |
| --- | --- | --- | --- | --- |
| translational subunit | IHC expressed subunit |  | Mean CS (SD) | Significance |
| eIF5A | eIF5A normal stroma | BLT (n=3) | 8 (2.3) | U=39.0; p<.528 |
|  |  | control (n=32) | 8 (2.6) |  |
|  |  | EOC (n=57) | 4 (1.9) | U=304.0; p<.000 |
|  |  | control (n=32) | 8 (2.6) |  |
|  | eIF5A nuclear | BLT (n=43) | 12 (2.2) | U=274.0; p<.000 |
|  |  | control (n=27) | 8 (2.8) |  |
|  |  | EOC (n=75) | 12 (3.4) | U=588.0; p<.000 |
|  |  | control (n=27) | 8 (2.8) |  |
|  | eIF5A cytoplasm | BLT (n=43) | 4 (2.7) | U=371.5; p<.009 |
|  |  | control (n=27) | 8 (2.6) |  |
|  |  | EOC (n=75) | 4 (2.6) | U=284.0; p<.000 |
|  |  | control (n=27) | 8 (2.6) |  |
| eIF5B | eIF5B normal stroma | BLT (n=1) | 3 | U=8.5; p<.410 |
|  |  | control (n=32) | 1 (2.2) |  |
|  |  | EOC (n=50) | 1 (1.3) | U=599.0; p<.046 |
|  |  | control (n=32) | 1 (2.2) |  |
|  | eIF5B nuclear | BLT (n=43) | 0 | N/A |
|  |  | control (n=32) | 0 |  |
|  |  | EOC (n=74) | 0 | N/A |
|  |  | control (n=32) | 0 |  |
|  | eIF5B cytoplasm | BLT (n=43) | 4 (2.7) | U=395.5; p<.007 |
|  |  | control (n=32) | 0 (3.6) |  |
|  |  | EOC (n=74) | 1 (1.9) | U=978.5; p<.467 |
|  |  | control (n=32) | 0 (3.6) |  |
| eIF6 | eIF6 normal stroma | BLT (n=1) | 2 | U=0; p<.000 |
|  |  | control (n=32) | 4 (0.0) |  |
|  |  | EOC (n=75) | 2 (1.0) | U=160.0; p<.000 |
|  |  | control (n=32) | 4 (0.0) |  |
|  | eIF6 nuclear | BLT (n=43) | 0 | U=0; p<.000 |
|  |  | control (n=25) | 4 (1.9) |  |
|  |  | EOC (n=75) | 0 | U=0; p<.000 |
|  |  | control (n=25) | 4 (1.9) |  |
|  | eIF6 cytoplasm | BLT (n=43) | 8 (2-7) | U=349.5; p<.009 |
|  |  | control (n=25) | 4 (2.0) |  |
|  |  | EOC (n=75) | 6 (2.7) | U=793.5; p<.201 |
|  |  | control (n=25) | 4 (2.0) |  |
| eIF2G | eIF2G normal stroma | BLT (n=43) | 4 (2.5) | U=90.0; p<.721 |
|  |  | control (n=29) | 4 (1.3) |  |
|  |  | EOC (n=56) | 4 (1.9) | U=702.0; p<.126 |
|  |  | control (n=29) | 4 (1.3) |  |
|  | eIF2G nuclear | BLT (n=43) | 8 (3.0) | U=525.0; p<.169 |
|  |  | control (n=29) | 8 (0.7) |  |
|  |  | EOC (n=75) | 4 (2.5) | U=542.5; p<.000 |
|  |  | control (n=29) | 8 (0.7) |  |
|  | eIF2G cytoplasm | BLT (n=43) | 8 (2.7) | U=496.5; p<.076 |
|  |  | control (n=29) | 8 (0.7) |  |
|  |  | EOC (n=75) | 4 (2.3) | U=513.5; p<.000 |
|  |  | control (n=29) | 8 (0.7) |  |
| eIF2α | eIF2α normal stroma | BLT (n=3) | 3 (3.6) | U=35.0; p<.384 |
|  |  | control (n=32) | 4 (2.0) |  |
|  |  | EOC (n=69) | 3 (1.6) | U=509.5; p<.000 |
|  |  | control (n=32) | 4 (2.0) |  |
|  | eIF2α nuclear | BLT (n=43) | 0 | N/A |
|  |  | control (n=26) | 0 |  |
|  |  | EOC (n=75) | 0 | N/A |
|  |  | control (n=26) | 0 |  |
|  | eIF2α cytoplasm | BLT (n=43) | 8 (2.3) | U=176.0; p<.000 |
|  |  | control (n=26) | 4 (2.0) |  |
|  |  | EOC (n=75) | 8 (2.4) | U=317.0; p<.000 |
|  |  | control (n=26) | 4 (2.0) |  |
| eEF1A1 | eEF1A1 normal stroma | BLT (n=3) | 6 (3.6) | U=30.0; p<.272 |
|  |  | control (n=32) | 4 (4.9) |  |
|  |  | EOC (n=54) | 3 (2.7) | U=775.5; p<.420 |
|  |  | control (n=32) | 4 (4.9) |  |
|  | eEF1A1 nuclear | BLT (n=43) | 0 | N/A |
|  |  | control (n=23) | 0 |  |
|  |  | EOC (n=75) | 0 | N/A |
|  |  | control (n=23) | 0 |  |
|  | eEF1A1 cytoplasm | BLT (n=43) | 8 (2.1) | U=295.0; p<.004 |
|  |  | control (n=23) | 4 (3.4) |  |
|  |  | EOC (n=75) | 8 (2.7) | U=502.0; p<.001 |
|  |  | control (n=23) | 4 (3.4) |  |
| CS – composite score, IHC – immunohistochemistry, eIF – eukaryotic initiation factor, eEF – eukaryotic elongation factor, EOC – epithelial ovarian cancer, BLT – borderline tumour | | | | |

Supplemental Data Table 2

| Supplemental table 2: Determination of expression level cut-offs in epithelial ovarian cancer | | | | |
| --- | --- | --- | --- | --- |
| translational subunit | IHC expressed subunit | Mean (SD) | Low score | High score |
| eIF2α | eIF2α normal stroma | 3 (1.6) | < 3 | ≥ 3 |
|  | eIF2α cancer stroma | 3 (1.1) | < 3 | ≥ 4 |
|  | eIF2α nuclear | 0 | 0 | 0 |
|  | eIF2α cytoplasm | 9 (2.4) | < 9 | ≥ 9 |
| eEF1A1 | eEF1A1 normal stroma | 3 (2.7) | < 3 | ≥ 3 |
|  | eEF1A1 cancer stroma | 4 (3.4) | < 4 | ≥ 4 |
|  | eEF1A1 nuclear | 0 | 0 | 0 |
|  | eEF1A1 cytoplasm | 9 (2.7) | < 8 | ≥ 8 |
| eIF2G | eIF2G cytoplasm | 6 (2.3) | < 8 | ≥ 8 |
|  | eIF2G normal stroma | 4 (1.9) | < 4 | ≥ 4 |
|  | eIF2G cancer stroma | 3 (1.9) | < 3 | ≥ 3 |
|  | eIF2G nuclear | 6 (2.5) | < 6 | ≥ 6 |
| eIF5A | eIF5A cytoplasm | 4 (2.6) | < 4 | ≥ 4 |
|  | eIF5A normal stroma | 4 (1.8) | < 4 | ≥ 4 |
|  | eIF5A cancer stroma | 4 (2.0) | < 4 | ≥4 |
|  | eIF5A nuclear | 11 (3.4) | < 11 | ≥ 11 |
| eIF5B | eIF5B cytoplasm | 2 (1.9) | < 2 | ≥ 2 |
|  | eIF5B normal stroma | 2 (1.3) | < 2 | ≥ 2 |
|  | eIF5B cancer stroma | 2 (1.5) | < 2 | ≥ 2 |
|  | eIF5B nuclear | 0 | 0 | 0 |
| eIF6 | eIF6 cytoplasm | 6 (2.7) | < 6 | ≥ 6 |
|  | eIF6 normal stroma | 2 (1.0) | < 2 | ≥ 2 |
|  | eIF6 cancer stroma | 2 (1.0) | < 2 | ≥ 2 |
|  | eIF6 nuclear | 0 | 0 | 0 |
| IHC – immunohistochemistry, eIF – eukaryotic initiation factor, eEF – eukaryotic elongation factor | | | | |

Supplemental Data Table 3

| Table: Correlation matrix of translational marker subunit expression in epithelial ovarian cancer | | | | | | | | | | |
| --- | --- | --- | --- | --- | --- | --- | --- | --- | --- | --- |
|  | eIF2α | | | eEF1A1 | | | eIF2G | | | |
|  | eIF2α normal stroma | eIF2α  cancer stroma | eIF2α cyto | eEF1A1 normal stroma | eEF1A1 cancer stroma | eEF1A1 cyto | eIF2G cyto | eIF2G normal stroma | eIF2G cancer stroma | eIF2G nuclear |
| eIF2α cytoplasm | **p<.043**  **r_s_=.250** | p<.489  r_s_=.081 | N/A | p<.692  r_s_=.055 | p<.098  r_s_=.196 | p<.314  r_s_=.118 | p<.013  r_s_=.285 | p<.168  r_s_=.194 | p<.125  r_s_=.179 | **p<.031**  **r_s_=.249** |
| eIF2α cancer stroma | p<.798  r_s_=.032 | N/A | p<.043  r_s_=.250 | p<.737  r_s_=-.047 | p<.727  r_s_=-.042 | **p<.012**  **r_s_=-.288** | p<.782  r_s_=.032 | p<.352  r_s_=-.132 | p<.448  r_s_=.089 | p<.848  r_s_=.023 |
| eIF2α normal stroma | N/A | p<.798  r_s_=.032 | p<.043  r_s_=.250 | p<.820  r_s_=-.034 | p<.676  r_s_=.053 | p<.485  r_s_=.087 | p<.080  r_s_=.217 | p<.271  r_s_=.137 | p<.472  r_s_=-.106 | p<.187  r_s_=.165 |
| eEF1A1 normal stroma | p<.820  r_s_=-.034 | p<.737  r_s_=-.047 | p<.692  r_s_=.055 | N/A | p<.848  r_s_=-.027 | p<.086  r_s_=.236 | p<.247  r_s_=-.160 | p<.720  r_s_=.056 | p<.485  r_s_=.097 | p<.188  r_s_=-.182 |
| eEF1A1 cancer stroma | p<.676  r_s_=.053 | p<.727  r_s_=-.042 | p<.098  r_s_=.196 | p<.848  r_s_=-.027 | N/A | p<.289  r_s_=.127 | **p<.003**  **r_s_=.349** | p<.851  r_s_=-.027 | **p<.001**  **r_s_=.385** | **p<.016**  **r_s_=.282** |
| eEF1A1 cytoplasm | p<.485  r_s_=.087 | p<.289  r_s_=.127 | p<.314  r_s_=.118 | p<.086  r_s_=.236 | p<.289  r_s_=.127 | N/A | **p<.043**  **r_s_=.235** | p<.194  r_s_=.152 | **p<.024**  **r_s_=.261** | p<.194  r_s_=.152 |
| eIF2G cytoplasm | p<.080  r_s_=.217 | p<.782  r_s_=.032 | p<.013  r_s_=.285 | p<.247  r_s_=-.160 | **p<.003**  **r_s_=.349** | **p<.043**  **r_s_=.235** | N/A | p<.115  r_s_=.221 | **p<.006**  **r_s_=.316** | **p<.000**  **r_s_=.905** |
| eIF2G normal stroma | p<.271  r_s_=.137 | p<.352  r_s_=-.132 | p<.168  r_s_=.194 | p<.720  r_s_=.056 | p<.851  r_s_=-.027 | p<.194  r_s_=.152 | p<.115  r_s_=.221 | N/A | p<.748  r_s_=-.046 | p<.115  r_s_=.221 |
| eIF2G cancer stroma | p<.472  r_s_=-.106 | p<.448  r_s_=.089 | p<.125  r_s_=.179 | p<.485  r_s_=.097 | **p<.001**  **r_s_=.385** | **p<.024**  **r_s_=.261** | **p<.006**  **r_s_=.316** | p<.748  r_s_=-.046 | N/A | **p<.006**  **r_s_=.312** |
| eIF2G nuclear | p<.187  r_s_=.165 | p<.848  r_s_=.023 | **p<.031**  **r_s_=.249** | p<.188  r_s_=-.182 | **p<.016**  **r_s_=.282** | p<.194  r_s_=.152 | **p<.000**  **r_s_=.905** | p<.115  r_s_=.221 | **p<.006**  **r_s_=.312** | N/A |
| eIF5A cyto | p<.674  r_s_=-.053 | p<.287  r_s_=.125 | **p<.026**  **r_s_=.257** | p<.877  r_s_=.021 | p<.753  r_s_=-.038 | p<.218  r_s_=.144 | p<.399  r_s_=.099 | p<.381  r_s_=.124 | p<.874  r_s_=.019 | p<.813  r_s_=.028 |
| eIF5A normal stroma | p<.350  r_s_=.132 | p<.195  r_s_=-.174 | p<.145  r_s_=.196 | p<.654  r_s_=-.069 | p<.211  r_s_=.173 | p<.390  r_s_=.116 | **p<.029**  **r_s_=.289** | **p<.002**  **r_s_=.441** | **p<.008**  **r_s_=.346** | p<.775  r_s_=.039 |
| eIF5A cancer stroma | p<.841  r_s_=-.025 | p<.544  r_s_=.072 | **p<.002**  **r_s_=.361** | p<.350  r_s_=-.130 | **p<.005**  **r_s_=.330** | p<.268  r_s_=.130 | **p<.006**  **r_s_=.316** | p<.206  r_s_=.178 | **p<.011**  **r_s_=.294** | **p<.004**  **r_s_=.330** |
| eIF5A nuclear | p<.395  r_s_=-.106 | p<.481  r_s_=-.083 | p<.667  r_s_=.050 | p<.678  r_s_=.058 | p<.556  r_s_=.071 | **p<.026**  **r_s_=.258** | p<.630  r_s_=.057 | p<.234  r_s_=.168 | p<.106  r_s_=.188 | p<.515  r_s_=.076 |
| eIF5B  cyto | p<.923  r_s_=-.014 | p<.199  r_s_=.151 | p<.199  r_s_=.151 | p<.180  r_s_=.187 | p<.843  r_s_=.024 | p<.348  r_s_=-.111 | p<.139  r_s_=.173 | p<.782  r_s_=.040 | p<.343  r_s_=-.112 | p<.330  r_s_=.115 |
| eIF5B stroma normal | **p<.005**  **r_s_=.391** | p<.060  r_s_=-.235 | p<.060  r_s_=-.235 | p<.583  r_s_=-.085 | p<.931  r_s_=-.013 | p<.367  r_s_=.130 | p<.434  r_s_=.113 | p<.685  r_s_=-.064 | p<.600  r_s_=-.076 | p<.434  r_s_=.113 |
| eIF5B cancer stroma | p<.692  r_s_=-.057 | p<.724  r_s_=-.042 | p<.724  r_s_=-.042 | p<.546  r_s_=-.084 | p<.067  r_s_=.218 | p<.756  r_s_=-.037 | p<.226  r_s_=.143 | p<.395  r_s_=-.122 | p<.492  r_s_=.082 | p<.672  r_s_=.050 |
| eIF6 cyto | p<.570  r_s_=.071 | p<.221  r_s_=-.143 | p<.117  r_s_=.183 | p<.640  r_s_=-.065 | p<.893  r_s_=.020 | p<.189  r_s_=.153 | p<.286  r_s_=.125 | p<.318  r_s_=-.141 | p<.365  r_s_=.106 | p<.154  r_s_=.166 |
| eIF6 normal stroma | p<.227  r_s_=.169 | p<.674  r_s_=-.055 | p<.861  r_s_=-.023 | p<.893  r_s_=.020 | p<.499  r_s_=-.091 | p<.850  r_s_=-.025 | p<.642  r_s_=-.061 | p<.804  r_s_=.038 | p<.839  r_s_=-.027 | p<.783  r_s_=.036 |
| eIF6 cancer stroma | p<.106  r_s_=.201 | p<.760  r_s_=.036 | p<.095  r_s_=.194 | p<.493  r_s_=.082 | p<.493  r_s_=.082 | p<.236  r_s_=.138 | p<.779  r_s_=.033 | p<.430  r_s_=.112 | p<.065  r_s_=.214 | p<.342  r_s_=.111 |
|  | eIF6 | | | eIF5B | | | eIF5A | | | |
|  | eIF6 cyto | eIF6 normal stroma | eIF6 cancer stroma | eIF5B cyto | eIF5B normal stroma | eIF5B cancer stroma | eIF5A cyto | eIF5A normal stroma | eIF5A cancer stroma | eIF5A nuclear |
| eIF6 cyto | N/A | p<.573  r_s_=.074 | p<.541  r_s_=.072 | p<.122  r_s_=.181 | p<.270  r_s_=-.159 | p<.369  r_s_=-.107 | p<.215  r_s_=.145 | p<.865  r_s_=.023 | p<.467  r_s_=.086 | p<.304  r_s_=.120 |
| eIF6 normal stroma | p<.573  r_s_=.074 | N/A | **p<.000**  **r_s_=.715** | p<.616  r_s_=-.067 | p<.460  r_s_=-.110 | p<.866  r_s_=.023 | p<.518  r_s_=-.085 | p<.599  r_s_=.075 | p<.775  r_s_=-.038 | p<.092  r_s_=.220 |
| eIF6 cancer stroma | p<.541  r_s_=.072 | **p<.000**  **r_s_=.715** | **N/A** | p<.784  r_s_=.032 | p<.296  r_s_=.151 | p<.491  r_s_=.082 | p<.795  r_s_=.031 | p<.934  r_s_=.011 | p<.677  r_s_=.049 | **p<.009**  **r_s_=.299** |
| eIf5B cyto | p<.122  r_s_=.181 | p<.616  r_s_=-.067 | p<.784  r_s_=.032 | N/A | p<.767  r_s_=.043 | **p<.030**  **r_s_=.256** | **p<.007**  **r_s_=313** | **p<.041**  **r_s_=-.274** | p<.908  r_s_=.014 | p<.979  r_s_=.003 |
| eIF5B normal stroma | p<.270  r_s_=-.159 | p<.460  r_s_=-.110 | p<.296  r_s_=.151 | p<.767  r_s_=.043 | N/A | **p<.005**  **r_s_=.391** | p<.199  r_s_=-.185 | p<.811  r_s_=.038 | p<.539  r_s_=.089 | p<.888  r_s_=-.020 |
| eIF5B cancer stroma | p<.369  r_s_=-.107 | p<.866  r_s_=.023 | p<.491  r_s_=.082 | **p<.030**  **r_s_=.256** | **p<.005**  **r_s_=.391** | N/A | p<.629  r_s_=-.057 | p<.052  r_s_=-.261 | p<.577  r_s_=.067 | p<.238  r_s_=-.140 |
| eIF5A cyto | p<.215  r_s_=.145 | p<.518  r_s_=-.085 | p<.795  r_s_=.031 | **p<.007**  **r_s_=313** | p<.199  r_s_=-.185 | p<.629  r_s_=-.057 | N/A | p<.775  r_s_=.039 | **p<.006**  **r_s_=.318** | p<.226  r_s_=.141 |
| eIF5A normal stroma | p<.865  r_s_=.023 | p<.599  r_s_=.075 | p<.934  r_s_=.011 | **p<.041**  **r_s_=-.274** | p<.811  r_s_=.038 | p<.052  r_s_=-.261 | p<.775  r_s_=.039 | N/A | **p<.000**  **r_s_=.451** | p<.591  r_s_=.073 |
| eIF5A cancer stroma | p<.467  r_s_=.086 | p<.775  r_s_=-.038 | p<.677  r_s_=.049 | p<.908  r_s_=.014 | p<.539  r_s_=.089 | p<.577  r_s_=.067 | **p<.006**  **r_s_=.318** | **p<.000**  **r_s_=.451** | N/A | p<.230  r_s_=.141 |
| eIF5A nuclear | p<.304  r_s_=.120 | p<.092  r_s_=.220 | **p<.009**  **r_s_=.299** | p<.979  r_s_=.003 | p<.888  r_s_=-.020 | p<.238  r_s_=-.140 | p<.226  r_s_=.141 | p<.591  r_s_=.073 | p<.230  r_s_=.141 | N/A |
| eIF – eukaryotic initiation factor, eEF – eukaryotic elongation factor | | | | | | | | | | |
